# Socioeconomic inequality in life expectancy in India

**DOI:** 10.1136/bmjgh-2019-001445

**Published:** 2019-05-09

**Authors:** Miqdad Asaria, Sumit Mazumdar, Samik Chowdhury, Papiya Mazumdar, Abhiroop Mukhopadhyay, Indrani Gupta

**Affiliations:** 1 LSE Health, London School of Economics and Political Science, London, UK; 2 Centre for Health Economics, University of York, York, UK; 3 Ambedkar University Delhi, Delhi, India; 4 Department of Health Sciences, University of York, York, UK; 5 Economics and Planning Unit, Indian Statistical Institute Delhi Centre, New Delhi, India; 6 Health Policy Research Unit, Institute of Economic Growth, Delhi, India

**Keywords:** health inequality, life expectancy, socioeconomic inequality, India

## Abstract

**Introduction:**

Concern for health inequalities is an important driver of health policy in India; however, much of the empirical evidence regarding health inequalities in the country is piecemeal focusing only on specific diseases or on access to particular treatments. This study estimates inequalities in health across the whole life course for the entire Indian population. These estimates are used to calculate the socioeconomic disparities in life expectancy at birth in the population.

**Methods:**

Population mortality data from the Indian Sample Registration System were combined with data on mortality rates by wealth quintile from the National Family Health Survey to calculate wealth quintile specific mortality rates. Results were calculated separately for males and females as well as for urban and rural populations. Life tables were constructed for each subpopulation and used to calculate distributions of life expectancy at birth by wealth quintile. Absolute gap and relative gap indices of inequality were used to quantify the health disparity in terms of life expectancy at birth between the richest and poorest fifths of households.

**Results:**

Life expectancy at birth was 65.1 years for the poorest fifth of households in India as compared with 72.7 years for the richest fifth of households. This constituted an absolute gap of 7.6 years and a relative gap of 11.7 %. Women had both higher life expectancy at birth and narrower wealth-related disparities in life expectancy than men. Life expectancy at birth was higher across the wealth distribution in urban households as compared with rural households with inequalities in life expectancy widest for men living in urban areas and narrowest for women living in urban areas.

**Conclusion:**

As India progresses towards Universal Health Coverage, the baseline social distributions of health estimated in this study will allow policy makers to target and monitor the health equity impacts of health policies introduced.

**Key questions**

**What is already known?**

- It is well established that health inequalities exist across various social groups and in various disease areas in India.
- Reducing socioeconomic health inequalities constitutes a key objective of health policy in the country.

**What are the new findings?**

- This is the first study that characterises the socioeconomic distribution of health in India as measured by life expectancy at birth and in so doing quantifies health inequalities occurring across the lives of the Indian population.
- The study uses data from the fourth round of the National Family Health Survey, the most comprehensive health survey conducted in India to date.

**What do the new findings imply?**

- These findings act as a baseline measure of the level of inequality in lifetime health across the country that can be used to assess the relative extent that the health of different subgroups within the population is improving as India rolls out Universal Health Coverage.

## Introduction

Socioeconomic inequality in health is everywhere evident in India with the poor living shorter and sicker lives than their richer compatriots and often facing catastrophic and impoverishing out-of-pocket outlays for accessing healthcare.^1–4^ This is unsurprising in a country where healthcare is largely provided by the private sector (76%) and paid for out of pocket (67%)—a rich case study in the full spectrum of market failures that occur when provision of healthcare is left to a largely unregulated private sector.^5–11^

There are a wealth of studies describing socioeconomic inequality in health in India in terms of various access and process indicators as well as in terms of various disease specific outcome measures.^12–17^ What is missing, however, is a quantification of socioeconomic inequality in overall lifetime health, providing a holistic measure synthesising inequalities emerging across the life course.^18^ The objective of this study is to produce such a measure by describing the patterning of health by wealth in India and in so doing to estimate mortality probabilities and associated distributions of life expectancy at birth by wealth quintile in the population. These estimates will provide a useful benchmark in order to gauge the level of socioeconomic inequality in overall health in the country and to monitor how this changes over time.

As India embarks on the journey towards Universal Health Coverage (UHC), public expenditure on healthcare remains modest as compared with other similarly placed countries.^19 20^ This dearth of fiscal space for health expenditure brings trade-offs between the various dimensions of the UHC agenda: (1) widening access and hence reducing health inequalities; (2) reducing out of pocket costs and hence providing financial risk protection; and (3) increasing the range of healthcare services and hence improving health—into sharp focus.^21^ Methods for conducting equity informative economic evaluations of health policies have recently emerged to help inform healthcare prioritisation decisions for countries pursing UHC where reducing health inequality and improving financial risk protection are core objectives of health policy.^22–24^ The estimates produced in this study may serve to underpin such equity informative economic evaluations for India.

## Methods

### Data

This study builds on the life tables produced by the Sample Registration System (SRS) data for 2011–2015. The SRS in India is the largest demographic surveillance system in the world covering 1.7 million households and 7.6 million individuals. Operated by the Registrar General India working under the Ministry of Home Affairs, the SRS continues to be the main source of information on fertility and mortality both at the state and national levels.^25^ The SRS life tables provide age group and sex specific mortality rates at overall, rural and urban geographies at the national level as well as at state level for a subset of states. The SRS data, however, do not provide information on socioeconomic differences in mortality rates; for this information, we instead use the National Family Health Survey (NFHS) round 4 (2015/16).^26^

The NFHS is the Indian edition of the Demographic and Health Surveys (DHS) programme conducted in over 90 countries around the world. The latest round of NFHS collected data on 601 509 households across India. The household level questionnaire categorises households according to their wealth level based on a DHS wealth index that measures possession of certain assets and access to certain utilities.^27^ Respondents were asked a wide range of questions in the NFHS as part of which they provided details regarding deaths occurring in their households over the 3 years immediately preceding the survey. From this information, we were able to collect data on the 74 945 deaths reported in the dataset and produce age group and sex specific annual mortality rates by household wealth quintiles at overall, rural and urban geographies.

### Analysis

Where detail on one or more characteristics regarding the deaths was missing in NFHS (typically age at death), we allocate these uncategorised deaths across each of the (age) groups in the ratio of the deaths occurring with known characteristics. For example, if x% of deaths overall are missing details about the age at death, we inflate the number of known deaths in each age group by 100/(100-x). Of the 74 945 deaths overall in the dataset, 466 (0.6%) were missing details on one or more characteristic.

Given that the NFHS was not powered to calculate national level mortality rates, we limited our use of the NFHS mortality data to adjusting the official vital statistics data used to produce the SRS mortality rates. The adjustments we applied serve to account for the relative differences observed in NFHS by wealth quintile. To do this, we first calculated overall mortality rates for all wealth quintiles by age group, sex and geography from the NFHS using the prescribed NFHS sample weights. NFHS provides data about the total number of deaths over the 3-year period prior to the survey. We use this information to calculate the 3-year mortality rates and simply divide by three assuming constant mortality rates across the 3 years to derive annual mortality rates. We then calibrated each of these rates by multiplying them by the appropriate calibration factor required to make the mortality rate for the particular subgroup as calculated from NFHS match the mortality rates given for that subgroup in SRS. Finally, we applied these same subgroup level calibration factors to the mortality rates derived for each of the wealth quintiles within each subgroup in the NFHS data.

As an example, to calculate the calibration factor for females aged 55–60 years, living in rural households, we performed the following calculation:

$$C_{55-60,female,rural}=\frac{M_{55-60,female,rural}^{SRS}}{M_{55-60,female,rural}^{NFHS}}$$

where *M* signifies the mortality rate for the given subgroup from the specified dataset and *C* signifies the subgroup specific calibration factor. We then assumed this calibration factor to be constant across the wealth quintiles within this subgroup and applied it to mortality rates further disaggregated by wealth quintile from the NFHS to get our final wealth quintile specific mortality rates for use in our lifetables:

$$M_{55-60,female,rural,quintile_{i}}=C_{55-60,female,rural}\times M_{55-60,female,rulal,quintile_{i}}^{NFHS}$$

This was repeated for each age group for both sexes and across both rural and urban households. The wealth quintiles used in the analysis were specific to the level of geography, that is, rural specific wealth quintiles were used to generate the results for the rural population, urban specific wealth quintiles were used to generate the results for the urban population and overall wealth quintiles were used to produce the overall results for the country. Age groups used in the analysis followed those used in the SRS starting from <1, 1–4 and then 5 year age bands until the age of 84 years with a final age group capturing those 85 years and over.

The calibrated and adjusted mortality rates estimated were then used to calculate age group-specific mortality probabilities (typically denoted by the symbol ‘q’ in lifetables) split by sex, geography and wealth quintile. These probabilities are central to producing lifetables and calculating life expectancies. These probabilities also serve as key parameters in decision analytic modelling of health policies, allowing analysts to model remaining expected survival should fatal health events be avoided by health interventions being evaluated.

We used these estimated mortality probabilities to calculate life expectancy at birth by sex, geography and wealth quintile using the Chiang method.^28^ We also calculated simple absolute gap and relative gap inequality indices across wealth quintiles by geography and sex:

$$absolutegap=e_{0,quintile_{5}}-e_{o,quintile_{1}}$$

$$realtivegap=\frac{e_{0,quintile_{5}}-e_{o,quintile_{1}}}{e_{o,quintile_{1}}}$$

where $e_{0,quintile_{5}}$ represents life expectancy at birth in the richest fifth of households and $e_{0,quintile_{1}}$ represents life expectancy at birth in the poorest fifth of households. These simple indices were chosen due to their ease of calculation and interpretation by policy makers.

Analyses were conducted in Stata V.13 and Microsoft Excel 2016. Full code listings including results and calculations in spreadsheet form can be found online at https://github.com/miqdadasaria/india_life_expectancy_inequality

### Patient and public involvement

This study was based purely on administrative data taken from SRS and NFHS; patients were not involved in the study directly.

## Results

**Figure 1:**
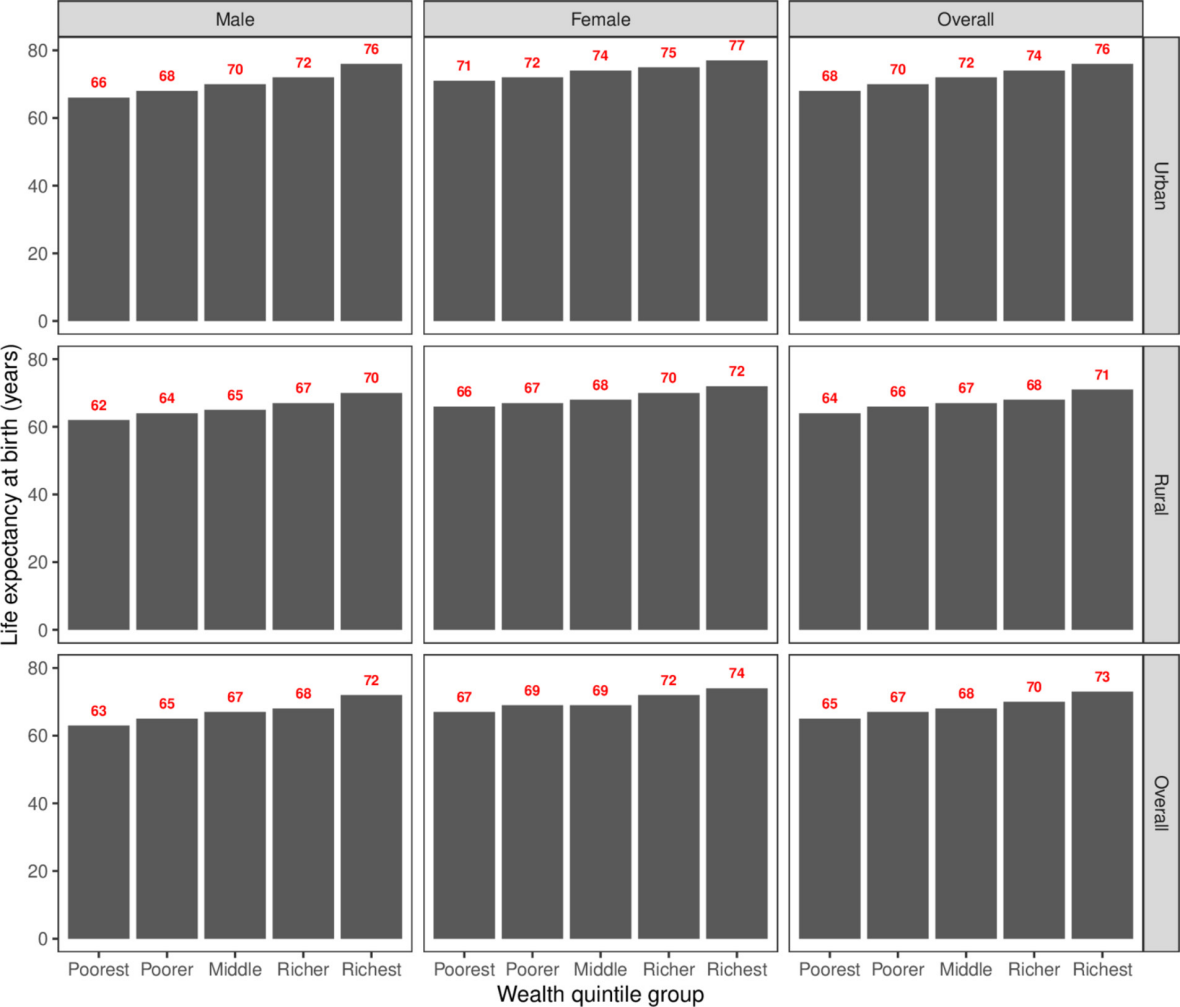
Distribution of life expectancy at birth for India calculated from SRS and NFHS-4.

The results given in table 1 show life expectancy at birth for men and women overall as well as in urban and rural areas specifically. Life expectancy is higher for women than for men and higher in urban areas than in rural areas. The distribution of life expectancy by wealth quintile depicted in figure 1 shows that life expectancy is lowest for men living in households from the poorest wealth quintile in rural areas at 62.2 years and highest for women living in households from the richest wealth quintile in urban areas at 77.0 years. A socioeconomic gradient in life expectancy is apparent within each of the subgroups examined with those living in wealthier households having higher life expectancies.

**Table 1:** Life expectancy at birth for India by sex, geography and wealth quintile

	Urban			Rural			Overall		
	*Male*	*Female*	*Overall*	*Male*	*Female*	*Overall*	*Male*	*Female*	*Overall*
***Wealth quintile***									
Q1 Poorest	66.4	70.8	68.4	62.2	65.9	64.0	63.2	67.1	65.1
Q2 Poorer	67.7	71.9	69.6	63.7	67.3	65.5	65.4	68.7	67.0
Q3 Middle	70.3	73.7	71.9	65.2	68.5	66.7	66.6	69.4	67.9
Q4 Richer	72.3	74.8	73.5	66.9	69.8	68.2	67.8	71.6	69.6
Q5 Richest	75.5	77.0	76.3	69.7	72.5	71.1	71.6	74.1	72.7
**Total**	**70.5**	**73.5**	**71.9**	**65.6**	**68.7**	**67.1**	**66.9**	**70.0**	**68.3**
***Inequality***									
Absolute: Q5-Q1	9.1	6.2	7.8	7.5	6.6	7.1	8.3	7.0	7.6
Relative: (Q5/Q1)−1	13.8%	8.8%	11.4%	12.1%	10.0%	11.0%	13.2%	10.5%	11.7%

*Note national wealth quintiles used for overall columns, national urban wealth quintiles for urban columns and national rural wealth quintiles for rural columns.

Inequalities in life expectancy by wealth quintile measured in both absolute and relative terms are wider for men than for women and are widest for men living in urban areas and narrowest for women living in urban areas. The average gap in life expectancy between people living in the richest quintile of households as compared with the poorest across the country is 7.6 years. In relative terms this implies that those living in the richest fifth of households have life expectancies 11.7% higher than those living in the poorest fifth of households. The mortality probabilities underpinning these results are given in the tables 2–4 detailing results for urban, rural and overall geographies, respectively.

**Table 2:** Mortality probabilities for those living in urban households in India

Age interval	Urban																	
	*Male*						*Female*						*Overall*					
	Poorest	Poorer	Middle	Richer	Richest	Total	Poorest	Poorer	Middle	Richer	Richest	Total	Poorest	Poorer	Middle	Richer	Richest	Total
0–1	0.0358	0.0279	0.0228	0.0205	0.0160	0.0258	0.0366	0.0328	0.0283	0.0273	0.0148	0.0290	0.0364	0.0301	0.0251	0.0236	0.0155	0.0273
1–5	0.0045	0.0046	0.0030	0.0030	0.0033	0.0038	0.0055	0.0044	0.0030	0.0023	0.0020	0.0036	0.0048	0.0044	0.0030	0.0027	0.0028	0.0037
5–10	0.0030	0.0027	0.0026	0.0030	0.0029	0.0028	0.0025	0.0036	0.0032	0.0018	0.0019	0.0027	0.0028	0.0032	0.0029	0.0025	0.0024	0.0028
10–15	0.0035	0.0036	0.0023	0.0018	0.0014	0.0026	0.0052	0.0031	0.0020	0.0014	0.0002	0.0026	0.0044	0.0033	0.0022	0.0016	0.0008	0.0026
15–20	0.0082	0.0038	0.0051	0.0021	0.0020	0.0044	0.0063	0.0049	0.0053	0.0051	0.0015	0.0048	0.0075	0.0042	0.0052	0.0032	0.0019	0.0046
20–25	0.0076	0.0083	0.0052	0.0054	0.0050	0.0064	0.0131	0.0030	0.0051	0.0033	0.0024	0.0054	0.0101	0.0058	0.0052	0.0043	0.0038	0.0059
25–30	0.0126	0.0104	0.0073	0.0056	0.0028	0.0078	0.0058	0.0085	0.0021	0.0044	0.0032	0.0048	0.0094	0.0095	0.0047	0.0050	0.0030	0.0063
30–35	0.0172	0.0131	0.0089	0.0090	0.0041	0.0104	0.0074	0.0075	0.0042	0.0035	0.0021	0.0048	0.0127	0.0105	0.0067	0.0063	0.0031	0.0078
35–40	0.0193	0.0226	0.0134	0.0099	0.0085	0.0148	0.0093	0.0035	0.0097	0.0079	0.0028	0.0066	0.0146	0.0133	0.0118	0.0092	0.0055	0.0108
40–45	0.0263	0.0253	0.0207	0.0154	0.0077	0.0189	0.0172	0.0120	0.0069	0.0055	0.0064	0.0092	0.0221	0.0192	0.0141	0.0107	0.0070	0.0143
45–50	0.0461	0.0349	0.0230	0.0218	0.0194	0.0282	0.0276	0.0164	0.0109	0.0113	0.0079	0.0140	0.0376	0.0260	0.0173	0.0167	0.0136	0.0214
50–55	0.0535	0.0498	0.0427	0.0341	0.0268	0.0399	0.0423	0.0362	0.0216	0.0189	0.0215	0.0265	0.0483	0.0435	0.0335	0.0272	0.0238	0.0336
55–60	0.0813	0.0791	0.0514	0.0590	0.0411	0.0591	0.0533	0.0557	0.0427	0.0395	0.0232	0.0408	0.0666	0.0669	0.0464	0.0500	0.0331	0.0501
60–65	0.1088	0.1068	0.0940	0.0778	0.0505	0.0838	0.0773	0.0580	0.0672	0.0599	0.0514	0.0621	0.0928	0.0838	0.0811	0.0694	0.0508	0.0734
65–70	0.1876	0.1384	0.1464	0.1051	0.0804	0.1254	0.1056	0.1186	0.0991	0.1094	0.0968	0.1056	0.1454	0.1279	0.1238	0.1069	0.0875	0.1157
70–75	0.2501	0.2353	0.2191	0.1754	0.1429	0.1986	0.1654	0.1864	0.1757	0.1487	0.1644	0.1680	0.2071	0.2100	0.1966	0.1629	0.1516	0.1832
75–80	0.2831	0.3824	0.3281	0.2651	0.2386	0.2919	0.2286	0.2934	0.2923	0.2839	0.2112	0.2614	0.2540	0.3378	0.3093	0.2745	0.2251	0.2760
80–85	0.3971	0.4789	0.4749	0.4793	0.3621	0.4350	0.3868	0.4475	0.4049	0.3266	0.4212	0.3970	0.3899	0.4597	0.4407	0.4049	0.3905	0.4152

Calculated using data from NFHS-4 (2015–2016) and SRS (2011–2015).

NFHS, National Family Health Survey; SRS, Sample Registration System.

**Table 3:** Mortality probabilities for those living in rural households in India

Age Interval	Rural																	
	*Male*						*Female*						*Overall*					
	Poorest	Poorer	Middle	Richer	Richest	Total	Poorest	Poorer	Middle	Richer	Richest	Total	Poorest	Poorer	Middle	Richer	Richest	Total
0–1	0.0607	0.0527	0.0452	0.0358	0.0279	0.0458	0.0660	0.0511	0.0448	0.0394	0.0277	0.0475	0.0631	0.0519	0.0451	0.0374	0.0279	0.0466
1–5	0.0102	0.0082	0.0079	0.0089	0.0048	0.0082	0.0186	0.0137	0.0119	0.0106	0.0078	0.0131	0.0140	0.0107	0.0099	0.0101	0.0062	0.0105
5–10	0.0042	0.0042	0.0044	0.0049	0.0052	0.0045	0.0037	0.0044	0.0047	0.0057	0.0055	0.0046	0.0039	0.0043	0.0045	0.0052	0.0053	0.0045
10–15	0.0054	0.0043	0.0030	0.0024	0.0033	0.0038	0.0047	0.0037	0.0031	0.0021	0.0018	0.0033	0.0051	0.0040	0.0031	0.0023	0.0026	0.0036
15–20	0.0076	0.0073	0.0049	0.0031	0.0048	0.0056	0.0079	0.0059	0.0055	0.0046	0.0047	0.0057	0.0077	0.0066	0.0051	0.0038	0.0047	0.0056
20–25	0.0141	0.0096	0.0079	0.0074	0.0059	0.0086	0.0107	0.0095	0.0082	0.0062	0.0042	0.0075	0.0123	0.0096	0.0081	0.0068	0.0050	0.0080
25–30	0.0139	0.0143	0.0116	0.0077	0.0062	0.0103	0.0119	0.0096	0.0095	0.0054	0.0044	0.0079	0.0128	0.0120	0.0107	0.0067	0.0053	0.0092
30–35	0.0189	0.0164	0.0140	0.0124	0.0079	0.0135	0.0124	0.0098	0.0094	0.0071	0.0061	0.0089	0.0155	0.0131	0.0118	0.0099	0.0071	0.0112
35–40	0.0266	0.0232	0.0165	0.0172	0.0114	0.0187	0.0150	0.0126	0.0105	0.0099	0.0077	0.0110	0.0208	0.0178	0.0136	0.0137	0.0095	0.0149
40–45	0.0332	0.0230	0.0264	0.0254	0.0205	0.0255	0.0206	0.0170	0.0166	0.0141	0.0098	0.0154	0.0273	0.0199	0.0216	0.0201	0.0155	0.0206
45–50	0.0456	0.0424	0.0386	0.0336	0.0261	0.0366	0.0260	0.0229	0.0198	0.0193	0.0185	0.0209	0.0363	0.0330	0.0295	0.0266	0.0222	0.0290
50–55	0.0648	0.0566	0.0504	0.0551	0.0468	0.0540	0.0531	0.0455	0.0378	0.0345	0.0257	0.0381	0.0591	0.0513	0.0446	0.0458	0.0371	0.0467
55–60	0.1038	0.0929	0.0932	0.0774	0.0617	0.0837	0.0571	0.0596	0.0539	0.0474	0.0419	0.0515	0.0778	0.0747	0.0720	0.0614	0.0515	0.0664
60–65	0.1290	0.1277	0.1312	0.1051	0.0836	0.1145	0.0910	0.1005	0.0850	0.0959	0.0727	0.0887	0.1100	0.1143	0.1095	0.1004	0.0782	0.1019
65–70	0.1871	0.1899	0.1824	0.1603	0.1384	0.1703	0.1358	0.1326	0.1448	0.1334	0.1092	0.1308	0.1606	0.1615	0.1635	0.1465	0.1241	0.1505
70–75	0.2582	0.2621	0.2420	0.2399	0.2168	0.2433	0.1787	0.2190	0.1941	0.1988	0.1769	0.1930	0.2188	0.2398	0.2174	0.2186	0.1963	0.2177
75–80	0.3271	0.3737	0.3563	0.3469	0.2895	0.3367	0.2367	0.2884	0.3259	0.2808	0.2581	0.2771	0.2808	0.3313	0.3397	0.3132	0.2725	0.3060
80–85	0.4869	0.4910	0.5110	0.5076	0.4424	0.4868	0.3775	0.4276	0.4403	0.4567	0.4211	0.4257	0.4330	0.4588	0.4753	0.4814	0.4311	0.4558

Calculated using data from NFHS-4 (2015–2016) and SRS (2011–2015).

NFHS, National Family Health Survey; SRS, Sample Registration System.

**Table 4:** Mortality probabilities across both urban and rural households in India

Age Interval	Overall																	
	*Male*						*Female*						*Overall*					
	Poorest	Poorer	Middle	Richer	Richest	Total	Poorest	Poorer	Middle	Richer	Richest	Total	Poorest	Poorer	Middle	Richer	Richest	Total
0–1	0.0572	0.0456	0.0374	0.0297	0.0243	0.0410	0.0622	0.0447	0.0408	0.0319	0.0227	0.0431	0.0594	0.0453	0.0390	0.0307	0.0238	0.0420
1–5	0.0088	0.0075	0.0075	0.0058	0.0049	0.0071	0.0152	0.0121	0.0095	0.0092	0.0049	0.0108	0.0116	0.0095	0.0087	0.0073	0.0052	0.0088
5–10	0.0040	0.0039	0.0043	0.0045	0.0038	0.0041	0.0035	0.0041	0.0049	0.0048	0.0037	0.0041	0.0037	0.0040	0.0045	0.0046	0.0037	0.0041
10–15	0.0054	0.0036	0.0021	0.0037	0.0017	0.0035	0.0052	0.0028	0.0029	0.0025	0.0012	0.0031	0.0053	0.0032	0.0025	0.0031	0.0015	0.0033
15–20	0.0083	0.0059	0.0045	0.0046	0.0027	0.0053	0.0079	0.0058	0.0057	0.0043	0.0029	0.0055	0.0081	0.0059	0.0051	0.0045	0.0028	0.0054
20–25	0.0126	0.0087	0.0078	0.0072	0.0047	0.0080	0.0106	0.0091	0.0082	0.0038	0.0036	0.0069	0.0116	0.0089	0.0080	0.0055	0.0042	0.0074
25–30	0.0156	0.0126	0.0097	0.0078	0.0043	0.0095	0.0113	0.0093	0.0066	0.0049	0.0037	0.0069	0.0133	0.0110	0.0083	0.0064	0.0040	0.0083
30–35	0.0187	0.0157	0.0132	0.0105	0.0069	0.0125	0.0115	0.0102	0.0078	0.0059	0.0034	0.0076	0.0149	0.0130	0.0107	0.0083	0.0052	0.0101
35–40	0.0248	0.0189	0.0186	0.0169	0.0096	0.0175	0.0130	0.0116	0.0094	0.0061	0.0089	0.0097	0.0190	0.0153	0.0141	0.0117	0.0092	0.0136
40–45	0.0305	0.0235	0.0272	0.0241	0.0134	0.0233	0.0176	0.0173	0.0159	0.0104	0.0078	0.0134	0.0244	0.0203	0.0219	0.0177	0.0107	0.0186
45–50	0.0442	0.0374	0.0373	0.0313	0.0234	0.0339	0.0242	0.0201	0.0197	0.0186	0.0132	0.0187	0.0347	0.0290	0.0287	0.0252	0.0184	0.0266
50–55	0.0593	0.0497	0.0490	0.0542	0.0390	0.0493	0.0459	0.0437	0.0359	0.0284	0.0233	0.0342	0.0525	0.0466	0.0430	0.0430	0.0318	0.0423
55–60	0.0893	0.0847	0.0752	0.0748	0.0622	0.0755	0.0563	0.0544	0.0545	0.0493	0.0316	0.0484	0.0709	0.0684	0.0639	0.0617	0.0477	0.0614
60–65	0.1220	0.1196	0.1067	0.1072	0.0762	0.1052	0.0864	0.0819	0.0931	0.0759	0.0682	0.0809	0.1043	0.1013	0.1000	0.0922	0.0723	0.0934
65–70	0.1826	0.1767	0.1591	0.1608	0.1143	0.1575	0.1233	0.1246	0.1355	0.1232	0.1138	0.1239	0.1528	0.1509	0.1470	0.1422	0.1135	0.1407
70–75	0.2443	0.2384	0.2510	0.2299	0.1911	0.2308	0.1743	0.2016	0.1996	0.1775	0.1783	0.1862	0.2095	0.2193	0.2251	0.2031	0.1837	0.2081
75–80	0.3174	0.3467	0.3529	0.3256	0.2882	0.3247	0.2348	0.2928	0.2755	0.2886	0.2715	0.2728	0.2752	0.3193	0.3135	0.3060	0.2788	0.2979
80–85	0.4647	0.4898	0.4918	0.4732	0.4461	0.4723	0.3895	0.4295	0.4509	0.4439	0.3745	0.4173	0.4276	0.4593	0.4702	0.4575	0.4104	0.4442

Calculated using data from NFHS-4 and SRS (2011–2015).

NFHS, National Family Health Survey; SRS, Sample Registration System.

These tables contain the mortality probabilities commonly referred to by the symbol ‘q’ in life tables. The probabilities in these tables can be used to construct life tables and associated life expectancies as well as to parameterise mortality probabilities in decision analytic models.

## Discussion

In most countries, under-5 mortality rates are higher in males than in females. Our study confirms the unusual result found in other recent studies, that for India under-5 mortality, rates are higher for females than for males.^29^ Despite this, life expectancy at birth for women was found to be higher than for men in every wealth quintile across both urban and rural households. The gender divide in life expectancy is a well-established result observed across the world and explained by a combination of behavioural and biological factors. It is reassuring to see this result confirmed at various levels of disaggregation in the Indian population and gives us some confidence that the adjustments that we have made for deriving socioeconomic distributions of life expectancy produce plausible results. However, the results regarding gender differences should be interpreted with caution given historical concerns about the greater degree of under-recording of female as compared with male mortality in the SRS.^30^

Life expectancy was found to be higher in urban households than in rural households for both men and women in every wealth quintile. This is largely driven by differences in under-5 mortality rates that were found to be much lower in urban areas than in rural areas as can be seen in the estimates of mortality probabilities reported in tables 2 and 3. This is consistent with the literature suggesting that as countries undergo epidemiological transition, the greater access to healthcare and better nutrition found in urban settings begins to outweigh the higher potential risk of catching infectious diseases in these more densely populated environments tilting childhood mortality rates in favour of urban populations.^31 32^

A smooth socioeconomic gradient in life expectancy at birth was observed for men as well as women in both urban and rural settings with health consistently improving with increases in wealth within every subgroup. The gap in life expectancy between the richest and poorest quintiles of households ranged between 9.1 years (13.8%) for men in urban households to 6.2 years (8.8%) for women in urban households. This socioeconomic gradient is now a well-established result in a number of countries around the world, and it is unsurprising to see this pattern also being clearly evident in India. A large literature discussing the social determinants of health has emerged to explore the reasons that such differences exist and persist.^33 34^

Socioeconomic inequality in life expectancy at birth was wider for men than for women with the gap being most pronounced in urban households. The estimates of mortality probability reported in tables 2–4 suggest that this is largely driven by the substantially lower female adult (between the ages of 20 and 50 years) mortality rates as compared with male adult mortality rates. This gap is wider for poorer households than for richer households and wider in urban households than in rural households. The relative shallowness of the socioeconomic mortality gradient for women may be explained by the substantial improvements in maternal mortality rates—improvements that have been more evident in urban than rural settings.^35 36^ With maternal mortality being a major driver of adult female mortality, particularly among the poor, these improvements have resulted in a reduction in socioeconomic inequality in female life expectancy and especially so in urban settings where better access to healthcare has improved the effectiveness of efforts to reduce maternal mortality. There have been no similar propoor developments in male mortality reduction, while poorer men are more likely to partake in risky health behaviours than their rich counterparts particularly in urban settings.^37 38^

This is the first study that we are aware of that calculates socioeconomic inequality in life expectancy at birth in India, thereby quantifying the degree of health inequality in the country in both a simple and yet comprehensive manner. This is also the first study to use the recently released NFHS dataset, the most comprehensive health survey conducted in India to date, to estimate health inequalities in the country. Similar studies in the UK and Ethiopia find inequalities in life expectancy at birth between the wealthiest and poorest quintiles of households of 6.5 years and 10 years, respectively, as compared with 7.6 years found for India in our study.^39 40^ These results suggest our estimates for India are plausible implying that in absolute terms health in India is less unequally distributed than in Ethiopia but more unequally distributed than in the UK, this ordering matches the latest estimates of income inequality as measured by the Gini index for the three countries calculated by the World Bank.^41^

Our study combines mortality data from national vital statistics with data for the Indian DHS dataset to produce socioeconomic distributions of life expectancy at birth. Similar datasets are available for many countries around the world, particularly for those countries pursing UHC for their populations. Our use of these standard datasets allows the methods described in the paper to be easily replicated in these countries.

Our study has a number of limitations. The first is that given the complexity of the analysis with multiple calibration steps and links across datasets, we have not been able to provide CIs for our estimates. Refining the methodology to capture the uncertainty in the estimated life expectancies and in the inequalities between these is a key area for further research. Second, we have provided results for inequality in life expectancy without adjusting for differential morbidity across the health quintiles. Ideally, we would have liked to estimate distributions of quality-adjusted life expectancy as measured in quality-adjusted life years or disability-adjusted life years.^42^ Developing credible methods for constructing such morbidity adjustments by wealth quintile for India, perhaps building on the disability adjustments used in the global burden of disease project, is another important area for further research.^43^ Analysis conducted in other countries suggests that inequalities widen once adjusted for morbidity differentials as poorer members of the population typically suffer greater levels of morbidity than their richer compatriots.^39^

Third, there have been concerns in the past about the data quality of both the SRS and the NFHS mortality data. Previous studies have found that, in the past (1980–2010), the SRS has under recorded deaths by approximately 11% in women and 4% in men.^30 44 45^ However, studies have found that on the whole mortality rates have been comparable between NFHS and SRS and can be relied on for ages between 0 years and 60 years.^46^ We were unable to find studies on the data quality of the latest round of the NFHS or the most recent years of SRS data. Our results should be interpreted in light of these data quality concerns. The fourth is that we have assumed that any differences between the SRS mortality rates and those calculated from NFHS are constant across wealth quintiles and so can be calibrated away using a wealth quintile independent calibration factor. It is possible that there may be systematically different reporting biases patterned by wealth quintile in the NFHS that we are unable to capture with our approach. Finally, our study focused on socioeconomic inequalities in health as patterned by wealth separately examined for men and women in rural and urban settings. However, various other population characteristics may also be associated with health disparities; in India, the most pertinent of these are caste and religion. Previous work on characteristics associated with relative age-specific mortality probabilities in India indicate that these other factors largely appear to be operating through their impact on socioeconomic conditions and that once socioeconomic status is controlled for they have limited residual effect on mortality.^47–50^

## Conclusion

It is evident that there are substantial socioeconomic health inequalities in India, if the country wants to tackle these then targeted policies with clear impacts on reducing health disparities should be identified and pursued. Monitoring health inequalities over time will help to determine whether such policies have been successful and will provide a first step towards understanding the determinants of these inequalities and the effectiveness of interventions in tackling them.
